# Chromosome segregation in *B. subtilis* is highly heterogeneous

**DOI:** 10.1186/s13104-020-05322-9

**Published:** 2020-10-09

**Authors:** Nina El Najjar, Peter L. Graumann

**Affiliations:** 1 LOEWE Center for Synthetic Microbiology, SYNMIKRO, Philipps Universität Marburg, Marburg, Germany; 2 Department of Chemistry, Philipps Universität Marburg, Marburg, Germany

**Keywords:** Bacterial cell cycle, Chromosome segregation, *Bacillus subtilis*, Single cell analyses

## Abstract

**Objective:**

The bacterial cell cycle comprises initiation of replication and ensuing elongation, concomitant chromosome segregation (in some organisms with a delay termed cohesion), and finally cell division. By quantifying the number of origin and terminus regions in exponentially growing *Bacillus subtilis* cells, and after induction of DNA damage, we aimed at determining cell cycle parameters at different growth rates at a single cell level.

**Results:**

*B. subtilis* cells are mostly mero-oligoploid during fast growth and diploid during slow growth. However, we found that the number of replication origins and of termini is highly heterogeneous within the cell population at two different growth rates, and that even at slow growth, a majority of cells attempts to maintain more than a single chromosome at all times of the cell cycle. Heterogeneity of chromosome copy numbers may reflect different subpopulations having diverging growth rates even during exponential growth conditions. Cells continued to initiate replication and segregate chromosomes after induction of DNA damage, as judged by an increase in origin numbers per cell, showing that replication and segregation are relatively robust against cell cycle perturbation.

## Introduction

The segregation of chromosomes is a central part of the cell cycle in all kinds of cells, yet poorly understood in bacteria [1, 2]. In a previous study, cell cycle parameters of *Bacillus subtilis* have been characterized by determination of population averages of DNA content and nucleoid morphology at four different growth rates. The pattern of increased numbers of replication forks at higher growth rates was similar compared to *Escherichia coli* cells: at low growth rates, *B. subtilis* was mostly mono or diploid (later in the cell cycle), meaning cells had one chromosome copy or two, while cells became mero-oligoploid at higher growth rates [3], meaning they contain several origin regions per chromosome copy because of overlapping round of replication. More recent publications have shown that the situation is different from what was calculated, namely that few cells are monoploid, even in poor growth medium [4], and that during exponential phase, most cells are mero-oligoploid in rich medium, and reduced chromosome copy numbers during entry into stationary phase [5].

In our recent study, we have shown that the pattern of chromosome segregation follows that of a linear, directed manner, and could be explained by entropy driven segregation based on diffusion [6]. In this work, we extend these analyses by scoring the number of origin or terminus regions, visualized by FROS, at two different growth rates, namely minimal and rich medium. We also characterized chromosome copy numbers by quantitative DAPI staining of the DNA, and investigated changes in the number of origins and termini after induction of DNA damage via Mitomycin C.

## Main text

### Polyploidy of *Bacillus subtilis*

We wished to determine the ploidy status of cells growing at a defined rate, and counted a) number of origins, b) of termini (stained by FROS), and c) number of chromosomes by quantitative DNA staining (DAPI intensity). We also used nucleoid morphology as a determinant for the number of chromosomes per cell, in combination with origin and terminus numbers. We used all four parameters because counting of FROS signals leads to an underestimation of copy numbers, since duplicated regions that are separated less than 250 nm will appear as one signal. All strains are described in [6] and were characterized in this prior work.

**Fig. 1:**
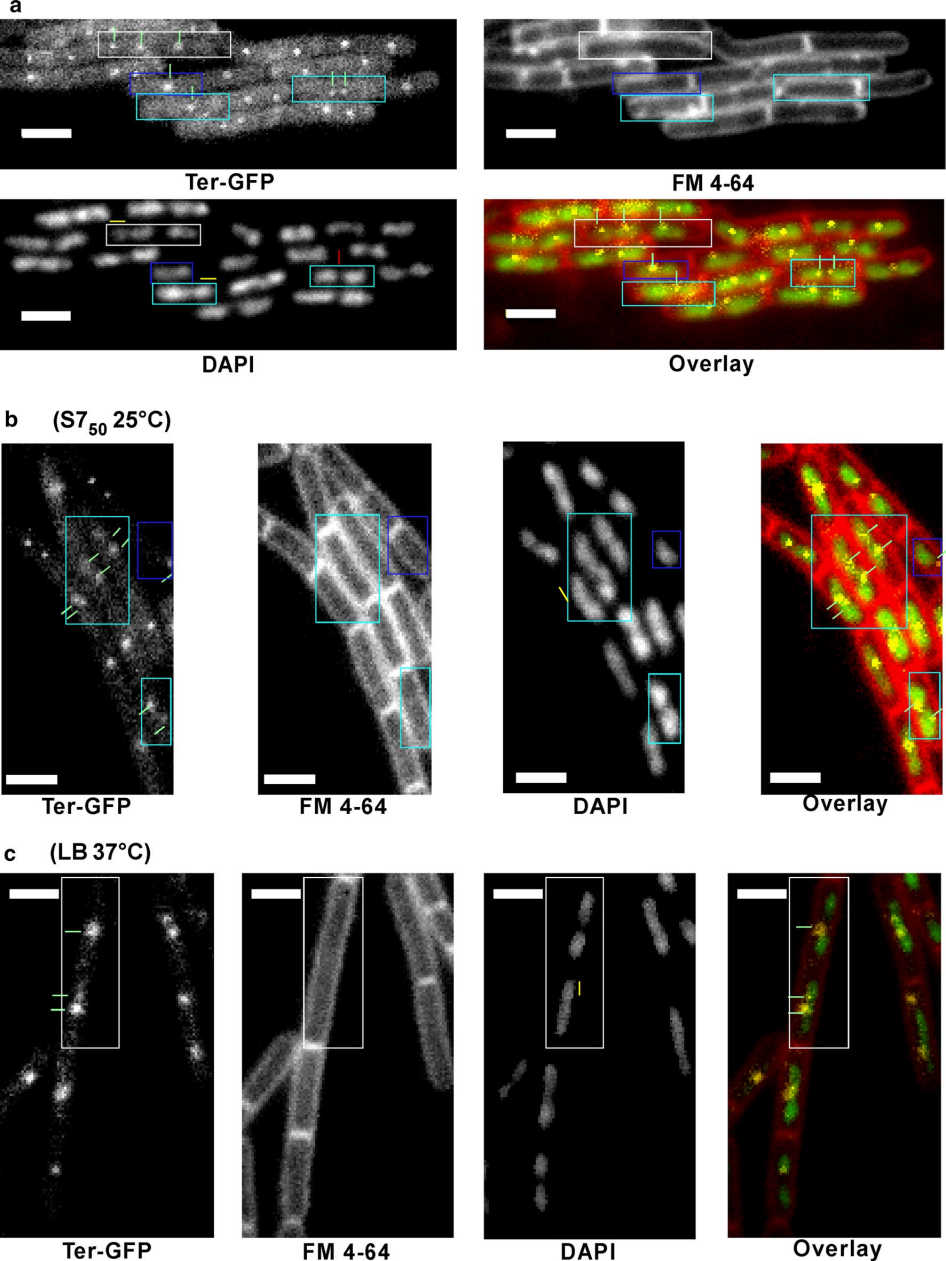
Exponentially growing cells of strain AT62 (terminus tag). **a** Example are shown of monoploid (dark blue rectangle), diploid (light blue rectangle), and polyploid cells (White rectangle). Green dashes point at the termini, the yellow dash represents 0.55 µm (the minimal length of a single nucleoid), and the red dash represents a distance of at least 3 pixels between 2 nuclei. **b** Cells grown in minimal medium at 25 °C: Cells are either diploid or monoploid. **c** Cells grown in LB at 37 °C. An elongated cell with 4 nucleoids is shown. White bars represent 2 µm

The numbers of origins and termini (see Fig. 1 for examples of imaging) were counted through visual scoring of signals per cell under conditions of slow growth in S7_50_ minimal medium (no amino acids) at 25 °C, with a doubling time of 90 min, or during fast growth at 37 °C in LB rich medium (doubling times of 26 min, similar to cells lacking a FROS system) at an OD_600_ ranging between 0.5 and 3, and the results are summarized in Table 1. As chromosomes can have multiple replication forks, and thus termini, we used the number of termini (and DAPI staining, see below) to determine the ploidity status of cells. The termini counts show that *Bacillus subtilis* is mostly diploid irrespective of the growth conditions, but that it is mero-polyploid in rich medium, where it can have multiple (up to 8) origins per cell (Table 1, first panel). The chromosome exists as a distinct, compact structure termed the ‘nucleoid’, for the entire cell cycle. Near the end of DNA replication, the nucleoid adopts a bi-lobed, dumb-bell shape that ultimately splits into two independent structures prior to cytokinesis [7]. In the terminus-tag strain, chromosomes forming a dumb-bell shape were counted as two nucleoids when they corresponded with two foci for the terminus tag. However, when only one focus was detected in the GFP (terminus tag) channel, a dumb-bell was considered as two nuclei when each lobe measured > 0.55 µm, or when the lobes were separated by at least 3 dark pixels. The offset of 0.55 was chosen through measuring several small separated lobes and averaging them. For the origin tag strain, the number of nucleoids was used for the classification of cells. Nucleoids with a dumb-bell shape were considered as one chromosome when the lobes were smaller than 0.7 µm and not separated by a minimum of 3 dark pixels. Cells with three or more distinct nucleoids were considered polyploid (Fig. 1), see also Fig. 1a in [6]. Elongated nucleoids occupying > 2.5 µm of the cell length were counted as 2 distinct chromosomes. The numbers of origins and termini were also counted every half an hour in in LB at 37 °C, starting from an OD_600_ of 0.1. Results are summarized in Table 1, second and third panels.

**Table 1:** Origins and termini count under slow and fast growth

	Slow growth (25 °C)		Fast growth (37 °C)	
	Foci/cell	n_t_	Foci/cell	n_t_
PG26 (origin tag)	1.8 ± 0.51	620	4.42 ± 1.49	848
AT62 (terminus tag)	1.49 ± 0.6	700	1.89 ± 0.66	848

PG26	Control 25 °C	1 h 37 °C	1.5 h 37 °C	2 h 37 °C	2.5 h 37 °C		3 h 37 °C
Percent polyploidy and average number of origins over time							
OD	0.7	0.11	0.3	0.7		1.8	2.5
% monoploid^a^	37.1	11	9	11		10	13
% diploid^b^	59.2	70	71	70		68	68
% polyploid^c^	3.7	19	20	19		22	19
Origins per cell	1.8 ± 0.51	4.8 ± 1.6	4.9 ± 2	4.9 ± 1.1		4.7 ± 0.55	4.1 ± 0.89
n_t_	620	300	300	350		400	400

AT62	Control 25 °C	1 h 37 °C	1.5 h 37 °C	2 h 37 °C	2.5 h 37 °C		3 h 37 °C
Percent ploididity and average number of origins over time							
OD	0.7	0.13	0.4	1		1.9	3
% monoploid^d^	35	17	13	12		15	12
% diploid^e^	61	74	72	71		69	75
% polyploid^f^	4	9	15	17		16	13
Termini per cell	1.49 ± 0.6	1.7 ± 0.5	1.93 ± 0.6	1.8 ± 1.1		1.72 ± 0.73	1.95 ± 0.8
n_t_	700	200	300	300		400	400

AT62	Control 25 °C	1.5 h 37 °C	2 h 37 °C	2.5 h 37 °C
Percent ploididity from automatically calculated average DAPI fluorescence				
OD	0.7	0.4	1	1.9
% monoploid	27.8	11.7	15	11.5
% diploid	67.5	68.3	63.3	62.25
% polyploid	4.6	20	21.7	26.25
n_t_	540	300	300	400

% polyploidy calculated over all time points at 37 °C = 23.65%.

*n_t_ refers to the total number of cells counted in each experiment

^a^1 or 2 origins

^b^2 to 4 origins

^c^4 to 8 origins

*n_t_ refers to the total number of cells counted in each time interval.

^d^1 terminus

^e^2 termini

^f^> 2 termini

Interestingly, the number of termini did not increase strongly at fast growth compared to slow growth, while the number of origins increased with growth rate, consistent with the observation that *B. subtilis* is diploid most of the time. However, the number of termini could be underestimated because in many cases it was difficult to tell if the focus between 2 nuclei corresponded to one or two termini. Based on the counted number of nuclei according to the above criteria, there was always a subset of polyploid cells under fast growth, but this subset never exceeded 22% for the origin tag strain (PG26), and 18% for the terminus tag (AT62) strain (Table 1). The total polyploidy calculated over all time points under fast growth conditions for both strains was 17.2% and 15.06%, respectively (Table 1, second and third panels). Fig. S1 (https://doi.org/10.6084/m9.figshare.12792407.v1) shows examples of monoploid, diploid, and polyploid cells. Most strikingly, under slow and even rapid growth conditions, fractions of monoploid, diploid and polyploidy cells were present, revealing a much higher heterogeneity of chromosome contents than previously anticipated.

### Estimation of the number of nuclei based on DAPI fluorescence

Although manual counting of terminus signals provided a good estimate on the polyploidy state of *B. subtilis*, it was prone to subjectivity and hence needed to be corroborated with an automated count. For this purpose, the ChainTracer software (Norbert Vischer, University of Amsterdam) was used together with the ImageJ plugin ObjectJ [8] to automatically calculate the amount of DAPI fluorescence in the cell.

The software can detect cell chains in a hyperstack, and extracts single cells from chains by detecting the septa and cell borders from channel containing the membrane- stain images. Integrated density of fluorescence (sum all of the pixels within a region) from the DAPI channel of the hyperstack is calculated, which quantifies the amount of DNA present in the cell. The terminus tag strain was chosen to perform the count under slow and fast growth. A threshold was specified for monoploid cells in each condition by averaging through fluorescence intensity of at least 30 cells measuring between 1.1 µm and 2 µm (i.e. the smallest cells) in each condition.

Average intensity of nucleoids in small cells was comparable in all counted samples. Monoploid cells were taken to have 1–1.69 genomes calculated from the threshold, diploid cells between 1.7 and 2.69, and polyploid cells to contain ≥ 2.69 genomes. Results are shown in Table 1, fourth panel. The percent polyploidy was underestimated by the manual count when compared to the automated based count. However, from both cases, it can be clearly deduced that *B. subtilis* is preferably diploid under slow and fast growth conditions, and that the number of polyploid cells is very small under slow growth and increases with the growth rate under fast growth. Cell length in minimal medium ranged between 1.894 µm and 4.112 µm, with an average of 3.05 ± 0.47 µm (n = 252). At faster growth both strains showed a direct correlation between the chromosomal content and the cell length whereby the polyploid cells were considerably longer than the diploid and the monoploid cells.

Thus, a *B. subtilis* culture contains cells with strongly diverging numbers of chromosomes and origin regions. This has to be taken into account when calculating cell cycle parameters from doubling times and replication periods. Interestingly, it has recently been shown that small RNAs induce diversity in transcription repressor AbrB levels generating heterogeneity in growth rates during the exponential growth phase. This leads to subpopulations of fast- and slow-growing *B. subtilis* cells, which has been proposed to increase fitness of the population when it has to deal with changes between favourable and unfavourable conditions [9].

### Effect of DNA double strand breaks on the polyploidy state of *B. subtilis*

In order to assess the effects of DNA double strand breaks on the chromosome count as well as the number of origins in *B. subtilis*, Mitomycin C (MMC) was added to a final concentration of 50 ng/ml to cells growing in minimal medium at 25 °C. In *B. subtilis*, MMC addition leads to a RecA-dependent transcriptional (SOS) response, and also to a RecA-independent response, which is mediated in part by initiator of replication protein DnaA [10]. MMC was shown to cause a relative increase of the dosage of genes proximal to the origin of replication relative to genes in the terminus region [10], which might be caused by re-initiation of replication in the absence of cell division, or in other words, because chromosome segregation and replication continue while cell division is blocked.

**Fig. 2:**
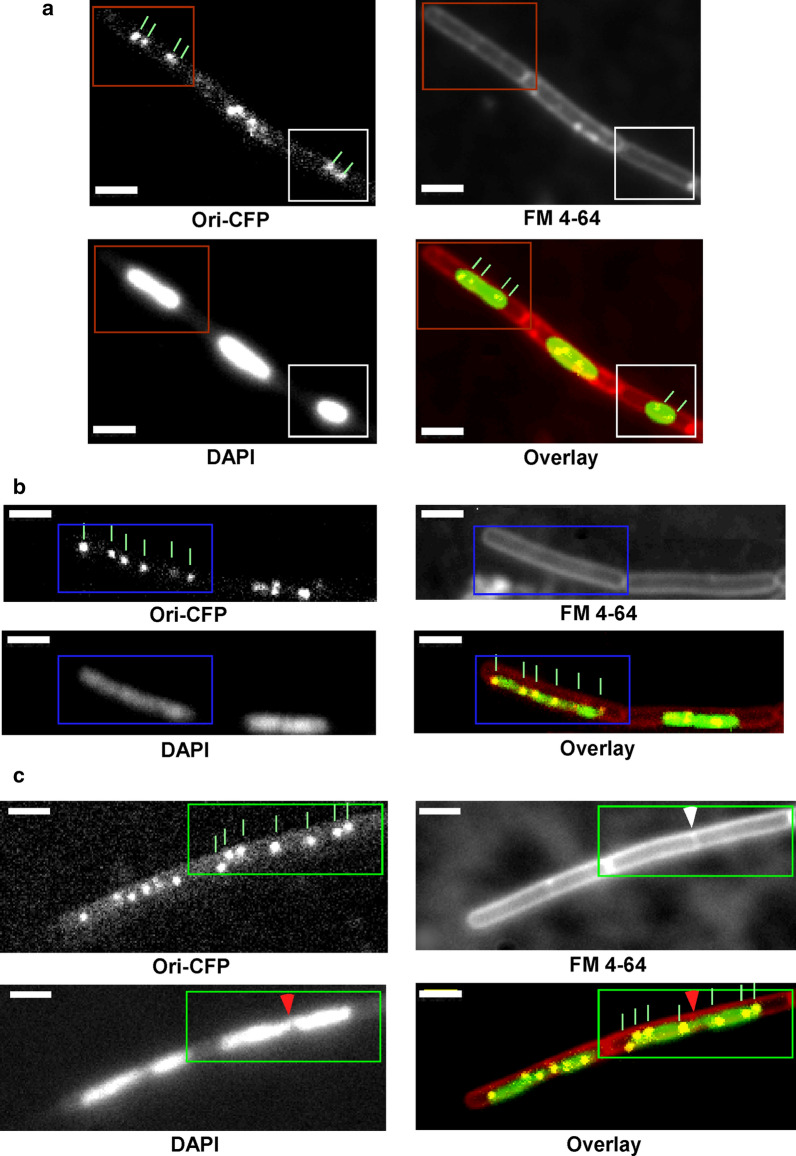
Cells of strain AT26 (origin tag) after DNA damage induction. **a** 30 min after addition of MMC, **b**, **c** 90 min after MMC addition. In **a** an example of one condensed nucleoid (white box) and fused nucleoids (red box) are shown. **b** An example of decondensed DNA in an elongated cell (green box), and **c** DNA bisected by a septum (green box). The red arrow points to the bisected nucleoid, while the white arrow shows the septum in question. Green lines point at the origins, of which up to seven can be seen in one cell in **b** White bars indicate 2 µm

We wanted to analyse the number of origins as well of termini in cells treated with MMC. Addition of MMC resulted in a strong reduction of growth for three hours. Cells imaged 30 min after MMC addition showed nucleoids with a condensed morphology (Fig. 2), and a majority of cells (~ 70% of 250 cells counted) contained one nucleoid instead of two, and 2.5 origins on average. The remaining cells had two nucleoids (Fig. 2). 90 min after MMC addition, around 15% of the cells were dead in each imaging field (as judged from high fluorescence throughout the cells), and were excluded from the analyses. The cells were elongated, average cell length increased from 2.87 ± 0.52 µm during exponential growth (n = 150 cells) to 4.4 ± 1.4 µm 90 min after MMC addition. Cell length fell in a range between 1.6 and 7.7 µm, with 20% of the cells being longer than 5 µm, revealing high heterogeneity. The number of origins increased from around 2 per cell prior to the induction of damage to 3.5 ± 1.4 (n = 200 cells). Of note, 16% of the MMC-treated cells had 5 origins or more, with a few having up to 8 origins, and a subset of the cells was anucleate (around 3%). 80% of cells longer than 5 µm contained an elongated, unsegregated nucleoid, and 0.5% of the cells had nucleoids bisected by a septum, evidence of chromosome segregation defects. The terminus tag strain showed comparable phenotypes, in that the cells were elongated and nucleoids were decondensed, 90 min after MMC addition. Excluding the dead cells, the average number of termini was 1.7 ± 0.6 (n = 224 cells), with 7.14% of the cells having 3 termini or even 4 in very rare cases.

These findings show that although MMC induces events of replication arrests due to interstrand crosslinks, and leads to atypical nucleoid morphology, most MMC-treated cells were still able to complete a replication cycle, or even reinitiated another cycle, giving rise to a double set of chromosomes after induction of DNA damage. The findings are in accordance with the report that MMC slows down the cell cycle considerably but does not stop it completely [6], and with findings from Goranov et al. showing that double strand breaks induced by MMC slow down the replication elongation beyond the origin proximal region [10].

## Limitations

As stated in reference [6], a small fraction of cells containing the FROS system showed irregularities in segregation. Cells were taken from exponential growth conditions and were imaged within 10 min, minimally influencing the actual number of origins and termini. Due to the resolution of light, origin or terminus regions separated by less than 250 nm would appear as a single signal, leading to underestimation of actual numbers.

## Data Availability

All data generated or analysed during this study are included in this published article. Raw images are available upon request at the corresponding author.

## Abbreviations

FROS: Fluorescent operator/repressor system
DAPI: 4′,6-Diamidin-2-phenylindol
